# Models and mechanisms of post-stroke dementia and cognitive impairment

**DOI:** 10.3389/fstro.2025.1563924

**Published:** 2025-05-09

**Authors:** Romeesa Khan, Patrick Devlin, Akihiko Urayama, Rodney M. Ritzel

**Affiliations:** ^1^Department of Neurology, McGovern Medical School, The University of Texas Health Science Center at Houston, Houston, TX, United States; ^2^University of Texas MD Anderson Cancer Center GSBS, Houston, TX, United States

**Keywords:** post-stroke cognitive impairment, dementia, chronic hypoperfusion, ischemic stroke, neurodegeneration

## Abstract

Stroke is a leading cause of death and disability globally, with significant long-term impacts such as post-stroke cognitive impairment (PSCI). PSCI affects up to one-third of stroke survivors, substantially increasing their risk of dementia, especially after recurrent strokes. Despite advances in acute stroke treatments, the mechanisms underlying PSCI remain poorly understood. Emerging evidence highlights that PSCI arises from a complex interplay of vascular damage, neurodegenerative pathologies, and chronic inflammation. This review explores the epidemiology and clinical characteristics of PSCI, emphasizing the role of age, education, vascular integrity, and comorbidities such as diabetes. Additionally, we examine experimental findings that utilize rodent models to elucidate the time course and biological mechanisms of PSCI. Notable contributions include insights from transgenic Alzheimer's disease (AD) mouse models, revealing how vascular and amyloid pathologies accelerate cognitive decline post-stroke. Moreover, studies on neuroinflammation and immune responses, such as those involving TREM2, underscore the significance of inflammatory pathways in PSCI. By integrating clinical and experimental findings, this literature review provides a comprehensive understanding of PSCI mechanisms, offering a foundation for developing targeted diagnostic tools and therapeutic interventions to mitigate the long-term cognitive effects of stroke.

## Introduction

**Figure 1:**
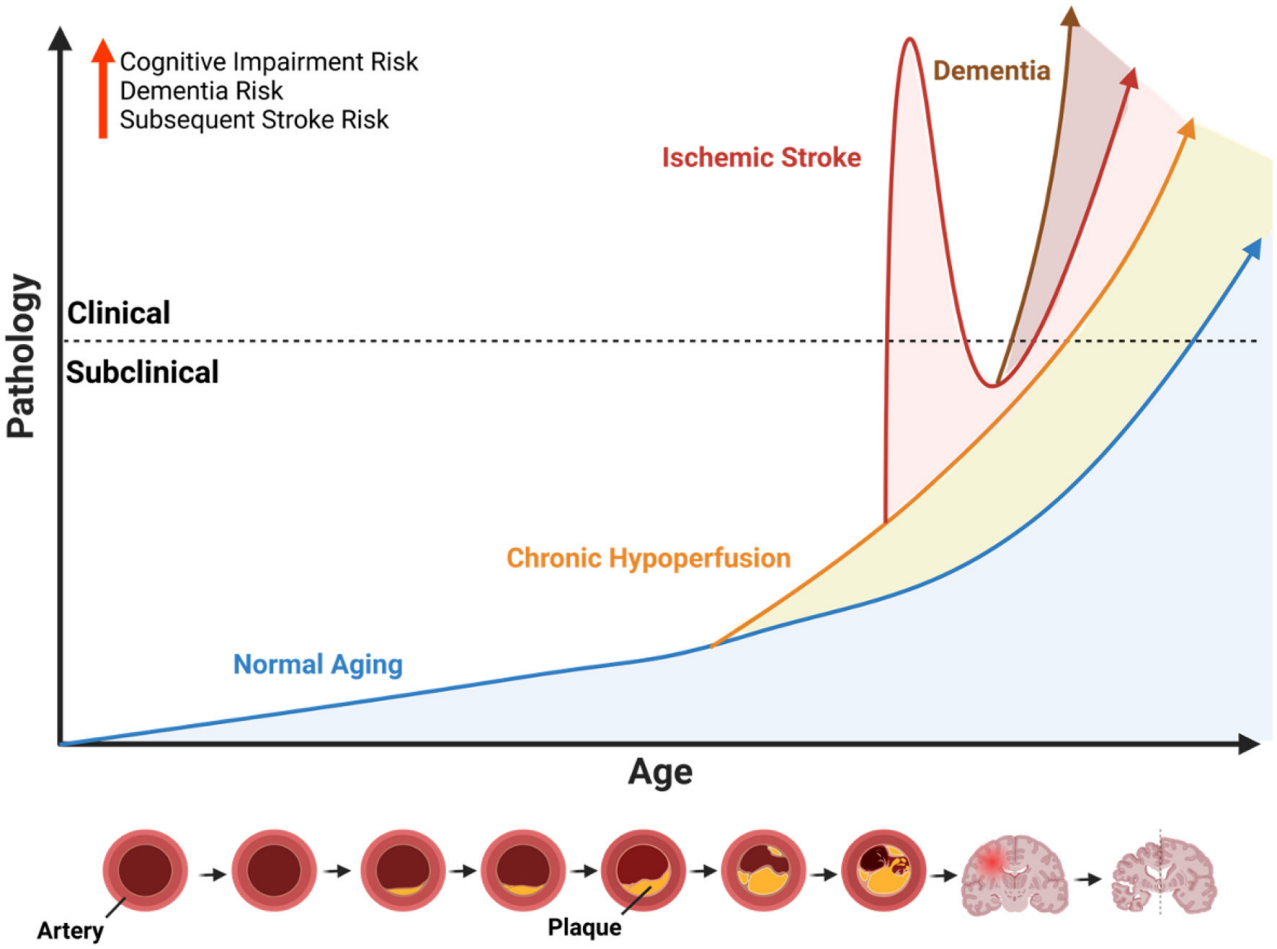
Influence of cerebrovascular disease on age-related neuropathology. Illustration of the conceptual framework to describe the potential age-accelerating effects of cerebrovascular disease. Because ischemic stroke is largely an age-related disease that primarily affects older individuals, the x-axis represents age as a function of time, and the y-axis represents age-related pathology. The dotted line indicates the threshold whereby subclinical pathology progresses to a point where it causes observable symptoms or measurable functional impairments (i.e., clinical). The blue line represents the slope of normal age-related pathology which increases over time. The orange line represents the age-accelerated pathology caused by chronic hypoperfusion due the narrowing of arteries caused by plaque accumulation (see diagram at the bottom). Chronic hypoperfusion independently accelerates the onset of acute ischemic and dementia, represented by the intersecting lines. The red line represents an acute ischemic stroke with immediate clinical features followed by a recovery period and overall age-accelerating effect on neuropathology and onset of dementia (brown line). Illustration created with BioRender.com.

Stroke is the fifth leading cause of death in the United States, and a leading cause of disability both in the United States and globally (Martin et al., 2024). While acute-phase treatments and research aimed at enhancing their effectiveness have contributed to a decline in overall stroke mortality (Goyal et al., 2016; Donato and Goswami, 2022), the enduring disability and cognitive impairment following a stroke remain to be further characterized. Almost one-fifth to one-third of all stroke survivors develop and experience some form of cognitive impairment and vascular dementia (VCID), and it has been shown that stroke survivors have up to an eighty-percent higher risk of developing dementia, with the risk almost tripling after recurrent stroke (Sachdev et al., 2006; Joundi et al., 2025; Guilbert, 2003). Moreover, this increased risk of dementia after stroke highlights a pressing need to identify effective strategies for early detection and intervention to mitigate cognitive decline and its associated impacts (Figure 1). Therefore, cognitive impairment post-stroke leaves a lasting burden on stroke survivors and their families, and is a significant public health issue. Although the pathological mechanisms underlying the acute inflammatory response following ischemic or hemorrhagic stroke, including potential targets for inhibition and the identification of acute inflammatory cell infiltration, have been largely established (Allen et al., 2012; Ali et al., 2000; Famakin, 2014), the mechanisms behind post stroke cognitive impairment (PSCI) remain to be elucidated. Emerging studies suggest that understanding the chronic effects of stroke on the brain—including changes in white matter integrity and synaptic plasticity—may offer additional insights into the progression of PSCI. There is a growing consensus of literature, both in the context of clinical findings and experimental research, that the advent of PSCI is a gradual process that is due to a multitude of factors. These factors include both vascular dysfunction as well as neurodegenerative pathologies that coincide with chronic inflammation post-stroke (Sun et al., 2014; Kim et al., 2022). Furthermore, factors such as pre-existing health conditions, lifestyle influences, and rehabilitation efforts may modify the risk and progression of PSCI, underscoring the need for a multidisciplinary approach to managing stroke recovery. To diagnose PSCI more effectively, develop therapeutic interventions, and target the chronic and persistent effects of stroke, we must first understand the secondary injury mechanisms underlying PSCI. In this review, we provide a brief overview of the epidemiology of PSCI, and summarize clinical and experimental findings in the context of post-stroke dementia.

## Epidemiology on post-stroke dementia and clinical findings

PSCI can be defined as cognitive impairment resulting from an ischemic or intracerebral hemorrhagic stroke (ICH), which can range from mild cognitive impairment to dementia (El Husseini et al., 2023). The time and course of PSCI incidence varies, with cognitive impairment and changes commonly starting to occur at 3–6 months poststroke, and worsening over time (Sun et al., 2014; Klimkowicz et al., 2002). However, in some cases, subtle cognitive deficits may be evident even earlier, within weeks post-stroke, highlighting the importance of early screening for cognitive changes. The risk and time of incidence for PSCI may be determined and influenced by a variety of factors, with previous studies showing that location of stroke (Rost et al., 2021), stroke severity (Elendu et al., 2023), or genetic preconditioning, such as carrying the APOE4 allele (Rajan et al., 2016), may all play a role in PSCI. For example, infarcts that occur in the left frontotemporal and right parietal lobes, and left thalamus, were strongly associated with PSCI (Weaver et al., 2021). Moreover, psychosocial factors such as depression, stress, and social isolation have been shown to exacerbate cognitive decline in stroke survivors, adding complexity to the clinical picture. Indeed, cognitive deficits due to stroke can overlap with other conditions like vascular dementia, Alzheimer's disease (AD), or post-stroke depression/fatigue can also affect cognition and are common post-stroke. Also, the majority (~80%) of patients with AD exhibit vascular amyloid aggregations leading to the development of cerebral amyloid angiopathy (CAA), which may cause further vascular complications including lobar hemorrhage (Brenowitz et al., 2015; Jäkel et al., 2022). Thus, multiple factors confound the specific mechanisms behind PSCI, making it difficult for clinicians to diagnose and for researchers to model, however, certain clinical findings may help to characterize and better understand the risk factors behind development of PSCI.

Older age, for example, is associated with accelerated cognitive decline post-stroke (Lo et al., 2022). Other factors, such as education level may also impact PSCI. In the NEDICES study (Contador et al., 2022), researchers found that low education and literacy were significantly associated with higher risk of post-stroke dementia, with low literacy levels and prior stroke incidence conferring significant risk to development or acceleration of PSCI. These findings suggest that cognitive reserve—a concept referring to the brain's resilience to neuropathological damage—may play a critical role in determining PSCI outcomes. Interventions aimed at enhancing cognitive reserve through lifelong learning and mental engagement may offer protective benefits (Weaver and Jaeggi, 2021). Additionally, other risk factors such as vascular structure and integrity, atrial fibrillation, and pre-existing conditions, such as diabetes, also influence PSCI (Drozdowska et al., 2020; Levine et al., 2023). Studies have found that type 2 diabetes existing prior to ischemic stroke occurrence, may lead to approximately a 2-fold increase in risk of PSCI compared to stroke survivors that do not suffer from this condition (Pendlebury and Rothwell, 2009; Shang et al., 2020). Similarly, hypertension, a leading modifiable risk factor, has been closely associated with both stroke incidence and subsequent cognitive decline, further highlighting the need for aggressive management of vascular risk factors in stroke patients (Faraco and Iadecola, 2013). The major clinical findings related to epidemiology of PSCI, in the context of characteristics associated with PSCI incidence and its risk factors, are summarized in Table 1.

**Table 1:** Epidemiological findings and clinical studies on PSCI in humans.

**References**	**Stroke type**	**Rate of PSCI**	**Stroke location**	**Experimental design**	**Pre-existing conditions/risk factors**	**Proposed mechanism**
Lo et al. (2019)	Ischemic Stroke	>33% after recurrent stroke	NA	Systematic review and meta-analysis	Multiple strokes, medial temporal lobe atrophy, female sex, family history of dementia	Stroke itself, multiple lesions, stroke characteristics
Zhou et al. (2024)	Ischemic Stroke	20% at 3 months post-stroke	Various locations	Prospective cohort study	Hypertension, diabetes, atrial fibrillation	Neuroinflammation, white matter changes
Zheng et al. (2019)	Hemorrhagic Stroke	15% at 6 months post-stroke	Basal ganglia	Retrospective cohort study	Chronic kidney disease, smoking	Blood-brain barrier disruption, oxidative stress
Delgado et al. (2022)	Ischemic Stroke	25% at 1 year post-stroke	Frontal lobe	Randomized controlled trial	Hyperlipidemia, sedentary lifestyle	Amyloid-beta accumulation, synaptic dysfunction
Lo et al. (2022)	Ischemic Stroke	30% at 2 years post-stroke	Parietal lobe	Cross-sectional study	Obesity, metabolic syndrome	Tau protein pathology, neuronal loss
Rajan et al. (2016)	Hemorrhagic Stroke	18% at 1 year post-stroke	Thalamus	Case-control study	Alcohol abuse, anticoagulant use	Cerebral microbleeds, iron deposition
Pendlebury and Rothwell (2009)	Ischemic Stroke	12% at 3 months post-stroke	Temporal lobe	Longitudinal study	Sleep apnea, depression	Hippocampal atrophy, cholinergic deficits
Shang et al. (2020)	Ischemic Stroke	22% at 6 months post-stroke	Occipital lobe	Observational study	Coronary artery disease, high homocysteine levels	Vascular endothelial dysfunction, reduced cerebral perfusion

## Experimental findings

The wide range of characteristics and risk factors associated with clinical PSCI findings make it difficult to fully study the occurrence of these post-stroke changes in the context of translational research. There is limited translational research and literature on the chronic effects of stroke, particularly the post-stroke period associated with accelerated cognitive decline, in laboratory models such as rodent models. However, more recent studies show that the course of PSCI development, in terms of time course, may be comparable between human and rodent models. In a study utilizing data from patients at a minimum of 5-months poststroke or longer, as well as a photothrombotic-stroke model in rats at 6-months poststroke, both the stroke survivors and rats display comparably impaired cognitive ability compared to controls (Chow et al., 2020). This finding contributes to the justification and validation of translational research in the field of PSCI and other long-term effects of stroke. Despite these advances, significant challenges remain, such as the limited ability of rodent models to fully replicate the complex human cognitive functions affected by stroke. Developing more sophisticated and human-relevant models, including non-human primate studies or organoid systems, may bridge this gap.

Although many biological factors may play a role in mediating PSCI, some have been identified as contributing significantly more toward risk and acceleration. Neurodegenerative factors and chronic brain inflammation, for example, may contribute to cognitive decline post-stroke. In one study, researchers showed that amyloid-PET positivity on scan chronically after stroke was associated with increased cognitive impairment scoring in individuals with stroke compared to age-matched controls (Godefroy et al., 2023). This finding and others like it (see Table 2), point toward a significant role for neurodegenerative pathology in the development of PSCI. Additionally, emerging evidence implicates tau pathology, in conjunction with amyloid deposition, in contributing to poststroke cognitive decline, suggesting overlapping mechanisms with other neurodegenerative diseases such as AD (Chi et al., 2019; Rangus and Bonilha, 2025). Here we look at how modeling of stroke in various rodent models of amyloid-related dementia may reveal specific mechanisms underlying the acceleration of dementia post-stroke.

**Table 2:** PSCI in experimental stroke models of AD and CAA.

**References**	**Stroke model**	**Disease model/strain**	**Time Post-stroke**	**Proposed mechanism**	**Key findings**
Osborne et al. (2024)	Transient MCAO	Cerebral amyloid angiopathy; Tg-SwDI transgenic mice	1 month	Microvascular dysfunction impairs hippocampal neurogenesis via altered PI3K signaling	Post-stroke hippocampal neurogenesis is significantly reduced in the presence of cerebral amyloid angiopathy due to impaired PI3K signaling
Zhang et al. (2020)	Micro-vessel occlusions induced by femtosecond laser	APP/PS1 transgenic mice	1-4 days	Microvascular lesions alter Aβ deposition and clearance	Microvascular lesions can alter the deposition and clearance of Aβ and confirm that Aβ plaques are dynamic structures
Marinescu et al. (2017)	Cerebral microbleeds	APP23 transgenic mice	3-4 months	Anticoagulation effects on micro- and macrohemorrhage	Anticoagulation with warfarin or dabigatran for 3 to 4 months does not promote the formation of CMBs in aged APP23 mice
Garcia-Alloza et al. (2011)	Cerebrovascular lesions	APPswe/PS1dE9	7 days	Vascular lesions increase Aβ accumulation	Vascular lesions accelerate Aβ accumulation by locally increasing production or decreasing clearance of Aβ
Milner et al. (2014)	Focal cerebral ischemia	Tg2576 transgenic mice	3 days	CAA increases infarction susceptibility	NA
Nguyen et al. (2018)	Distal MCAO with hypoxia induced	hAPP-SL mice	12 weeks	Amyloid and tau deposition co-localize with myelin repair pathways	Increase in BACE1 could be causing an inadvertent cleavage of its alternative substrate, AβPP, resulting in greater Aβ seeding and pathogenesis

## Importance of BBB regulation

The blood brain barrier (BBB) is mainly made up of vascular endothelial cells expressing tight junctions, low rates of transcytosis, and extremely limited fenestrations, which protects brain cells from direct exposure to circulating factors (Zlokovic, 2008; Montagne et al., 2017; Sweeney et al., 2018; Erickson and Banks, 2013; Erickson et al., 2012; Hashimoto et al., 2023; Kadry et al., 2020; Daneman and Prat, 2015; Sweeney et al., 2019; Banks, 2016; Hawkins and Davis, 2005). Barrier properties are not intrinsic to brain endothelial cells, as they require active induction and maintenance from other brain cells and extracellular matrix (ECM) proteins (Cucullo et al., 2011; Luissint et al., 2012; Baeten and Akassoglou, 2011; Ayloo et al., 2022; Howe et al., 2020). It also receives hormonal regulation such as estrogen which mediates the expression levels of claudin-5, one of the essential barrier-forming claudins in vascular endothelial cells (Erickson et al., 2012; Hashimoto et al., 2023; Burek et al., 2010).

Recent studies indicate that ECM abnormalities are a hallmark of many neurodegenerative diseases (Damodarasamy et al., 2020; Bonneh-Barkay and Wiley, 2009). Vascular events clearly alter the compositional expression of ECM proteins which affects the perivascular clearance of waste products generated in the inflamed brain (Howe et al., 2018, 2019). Alteration of the ECM in AD induces BBB disruption, and the resulting vascular leakage further remodels the ECM, creating a vicious cycle of cerebrovascular deterioration (Damodarasamy et al., 2020; Bonneh-Barkay and Wiley, 2009). Multiple studies found that the loss of BBB integrity precedes cognitive decline and neurodegeneration in AD (Erickson and Banks, 2013; Nation et al., 2019; Takechi et al., 2017; Hussain et al., 2021). Thus, maintaining barrier functions at early preclinical stages of AD may prevent or slow the progression to clinical AD, allowing additional opportunities for other prophylactic/early interventions (Yamazaki et al., 2019).

## Experimental stroke in AD/CAA

In recent studies on the chronic effects of stroke in rats, researchers found that the inflammatory phenotypes and structural brain changes that occur with stroke are persistent in the long-term (Ermine et al., 2021; Syeda et al., 2022). These studies, however, utilize models of stroke that are not fully translatable, or produce smaller, localized infarcts. They also do not include the study of stroke in genetic rodent models of neuropathological diseases, such as proteinopathies. Although future stroke studies in models of neurodegeneration, in the *chronic* phase of stroke, are needed to uncover the mechanisms behind PSCI, there are several studies that investigate PSCI in amyloid models in the more acute and subacute phases poststroke. In triple (3xTg-AD) and double (2xTg-AD; APPswe/PS1dE9) transgenic mouse models of AD, often used to model accelerated deposition of amyloid plaques in rodents, induction of an ischemic stroke by a permanent middle cerebral artery (MCA) occlusion demonstrated increased inflammation-associated accelerated aging phenotypes and promotion of rarefaction in AD mice with a stroke, compared to stroked wild-type controls (Zhang et al., 2019). In the hAPP-SL transgenic mouse model of AD, which expresses the human amyloid precursor protein (hAPP), Nguyen et al. found increases in both amyloid-beta plaque deposition as well as tau pathology in brains of mice with a distal MCA stroke-hypoxia model (Nguyen et al., 2018). Vascular pathology is also a common feature of advanced AD, with CAA and microvascular changes commonly observed in genetic mouse models, such as the Tg-ArcSwe model (Skaaraas et al., 2021). In terms of the relation between vascular integrity, or vascular damage, and amyloid deposition, studies show that constriction of blood flow to the brain may induce accelerated amyloid deposition. In a study utilizing a mouse model of CAA (Tg-SwDI) which predominantly shows capillary manifestations, with induced hypoperfusion, researchers found that CAA mice with 12 weeks of hypoperfusion had higher amounts of leptomeningeal amyloid deposits, as well as cortical microinfarcts, when compared to wild-type control mice (Okamoto et al., 2012). The acceleration of amyloid deposition and presence of cortical and other pathological brain changes points toward a mechanism of vascular integrity and inflammation as being related to increases in amyloid. This may imply that poststroke changes, such as vascular changes and other neurodegenerative pathology, contribute significantly to the development of PSCI.

## Experimental stroke with TREM2-mediated neuropathology

Persistent neuroinflammation, in addition to neurodegenerative changes, have also been studied as contributing factors of PSCI. Acute neuroinflammation following stroke is well-characterized (Alsbrook et al., 2023), with microglial and other immune cell infiltration being key drivers of this secondary injury, and is increasingly implicated as a chronic driver of stroke pathology (Goodman et al., 2023). TREM2, or triggering receptor expressed on myeloid cells-2, is a receptor for beta-amyloid that has been previously implicated in AD and phagocytosis of apoptotic debris, as well as regulation of microglial function (Zhao et al., 2018). TREM2 was identified as a significant contributing factor in a multitude of genome-wide studies (GWAS) (Gratuze et al., 2018), and therefore, is a major target in the context of understanding the mechanisms behind dementia-related pathologies, and may also be implicated in PSCI. In a study utilizing a TREM2-knockout (TREM2-KO) mouse model, KO mice that received a stroke (MCAO) displayed significantly decreased levels of inflammatory cytokines such as TNF, as well as decreases in gliosis, as demonstrated by lower expression of microglial markers in immunohistochemistry of brain tissue (Sieber et al., 2013). Although these studies further enhance our understanding on the role of TREM2, and related immunological mechanisms, in the context of dementia, it remains to be studied how TREM2 inhibition or global knockouts of TREM2 would affect poststroke pathology in the chronic phase; and whether this should be targeted in the acute phase of stroke, pertaining to acute inflammation, or as a delayed therapeutic intervention. Together, these findings shows that the interplay between immune-, non-immune-mediated inflammation, and neurodegenerative pathology such as amyloid deposition, all play a role in PSCI.

## Apoe-mediated PSCI in experimental stroke

In addition to proteinopathies and immune-mediated mechanisms of cognitive impairment, genetic factors also play a significant role in the development of dementia. Apolipoprotein E (ApoE), for example, is known to be the most prevalent of these genetic risk factors, as it is implicated in almost half of all known AD cases (Raulin et al., 2022). Having two copies of the ε4 allele of ApoE, ApoE4, has been shown to significantly increase risk for AD (Fortea et al., 2024). The role of APOE status in the context of PSCI has shown that APOE4 accelerates the development of dementia following stroke (Montagne et al., 2020). Experimental stroke research involving human APOE4 knock-in (hAPOE4 KI) mice aims to investigate the effects of this key genetic risk factor on stroke outcome. The hAPOE4 KI mouse model carrying the primary human variant of ApoE are selectively bred, allowing researchers to integrate human genetics and elucidate how this single variation of the gene alters the pathophysiology of stroke and long-term cognitive outlook (Montagne et al., 2020). In a study utilizing mouse models of three different human ApoE isoforms: ApoE2, ApoE3, and ApoE4, the authors found that infarct volumes and neurological deficit scores were significantly worsened after permanent MCAO in hAPOE4 KI mice compared to the other two isoform strains (Mori et al., 2003, 2004). Human ApoE was detected in neurons and astrocytes in the peri-infarct area, however, long-term outcomes were not assessed in these studies. Subsequent studies demonstrated that ApoE4 KI mice display an overall increase in ICH when compared to hAPOE3 KI mice, displaying increased vascular and parenchymal fibrillar amyloid deposits using thioflavin-S dye (Sullivan et al., 2008). In a subarachnoid hemorrhage model of stroke, hAPOE4 KI mice showed increased acute mortality, cerebral edema, functional deficits, and vasospasms when compared to hAPOE3 KI mice (Gao et al., 2006). To better understand the interactions between the molecular mechanisms of mixed vascular and AD dementia, researchers have subjected APOE4 KI:5XFAD transgenic mice to a subcortical white matter stroke by injecting the vasoconstrictive agent L-NIO. Surprisingly, L-NIO caused a significant reduction in amyloid plaque burden, improvements in cognitive performance, and an increase in the number and morphologic complexity of microglia compared with saline-injected control mice (Hayden et al., 2020). This study suggested that stroke may paradoxically promote amyloid clearance, potentially due to inducing a phagocytic state in microglia and opening of the blood-brain barrier. What role, if any, APOE4 played in this phenomenon is not clear. Further studies are needed to pinpoint the precise mechanistic role of the APOE4 in the development and initiation of post-stroke cognitive impairment, including investigation of the potentially protective APOE2 genotype on stroke sensitivity.

## Experimental stroke in mouse models of PD

In humans, Parkinson's disease (PD) is triggered by the aggregation of misfolded Alpha synuclein (α-synuclein) proteins that ultimately lead to neuron death due to loss of neuronal function. α-synuclein is a presynaptic protein that regulates synaptic vesicle trafficking and neurotransmitter release. PD is characterized by the abnormal aggregation and misfolding of α-synuclein, leading to the formation of Lewy bodies and Lewy neurites in affected neurons. Sudden loss or alteration in function of the α-synuclein gene can be experimentally manipulated in mice to address its role in stroke and its ability to aggravate Parkinsonism symptoms or accelerate its progression. In a seminal study by Lohmann et al., the effects of ischemic stroke were examined in young hemizygous TgM83^+/−^ mice overexpressing human α-synuclein with the familial A53T mutation. The authors induced transient 30-min ischemia via middle cerebral artery occlusion (MCAO) and FOLLOWED the mice for up to 14, 30, 90, 180, and 360 days post-injury (Lohmann et al., 2022). Stroke induced neuronal loss, astrogliosis, microgliosis, and motor dysfunction (in rotarod test) across all time points. Interestingly, microglial activation in the contralateral stroke hemisphere was evident with older age. These changes were seen alongside increased accumulation of α-synuclein and loss of dopaminergic neurons in the substantia nigra, a hallmark pathology of PD. These findings suggest that ischemic stroke may accelerate the onset and further exacerbate Parkinson's disease severity in predisposed individuals (Lohmann et al., 2022). A related study found that transient focal ischemia significantly upregulated serine-129 phosphorylation of α-synuclein and nuclear translocation. This important finding provides a direct link between stroke and the potential for abnormal aggregation of α-synuclein. Interestingly, they also examined the effects of α-synuclein KO and knockdown (KD) on experimental stroke outcome. It was observed that KO/siRNA mice showed reductions in mitochondrial fragmentation, oxidative stress, apoptosis, and autophagy. These attenuated molecular injury patterns coincided with increased functional motor recovery and smaller infarct volumes (Kim et al., 2016). Follow-up work from the same group assessed the effects of α-synuclein KD on stroke outcome as a function of age and sex. KD was achieved using siRNA that was administered intravenously at 30 min or 3 h reperfusion. KD mice showed improved motor function and decreased brain damage post-stroke in both adult and aged mice, regardless of sex (Chelluboina et al., 2020). Across both ages and sexes, reducing α-synuclein gene expression resulted in fewer motor deficits during the first week of stroke. However, in this study, young adult mice were subjected to 60-min MCAO vs. 35-min in aged mice which may affect the interpretation of these findings. Taken together, it is understood that α-Synuclein plays an important and direct role in mediating Parkinson's disease pathology, secondary brain injury, and PSCI.

## Conclusion/future perspectives

Post-stroke cognitive impairment (PSCI) represents a significant public health challenge, profoundly impacting the quality of life of stroke survivors and their families. Despite advances in stroke prevention and acute-phase treatments, the long-term cognitive consequences of stroke remain a critical area for research and clinical intervention. This review highlights the multifactorial nature of PSCI, encompassing vascular damage, neurodegenerative pathology, and persistent inflammation as interconnected contributors to cognitive decline. The clinical findings and experimental models discussed herein provide valuable insights into the complex pathophysiological mechanisms underlying PSCI. However, significant gaps remain in our understanding of how these mechanisms interact over time to drive cognitive impairment post-stroke. Future research should prioritize longitudinal studies that integrate clinical and experimental approaches to delineate the precise temporal dynamics and interactions between vascular injury, amyloid pathology, and inflammation. Advances in neuroimaging and biomarker development hold promise for early detection and monitoring of PSCI, enabling the identification of high-risk individuals and tailoring interventions accordingly.

Therapeutic strategies targeting chronic inflammation, vascular repair, and neurodegeneration offer potential avenues for mitigating PSCI. For example, anti-inflammatory agents and neuroprotective therapies, alongside interventions to enhance vascular integrity, merit further investigation in preclinical and clinical settings. Additionally, precision medicine approaches leveraging genetic and molecular profiling could inform personalized treatments, addressing individual risk factors such as APOE4 genotype or comorbid conditions like diabetes. The integration of interdisciplinary efforts, combining neurology, immunology, vascular biology, and neurodegeneration research, will be crucial in advancing our understanding of PSCI. By translating these findings into clinical practice, there is hope for improving outcomes for stroke survivors, reducing the burden of PSCI, and ultimately enhancing their long-term cognitive and functional recovery.
